# High accumulation of Mx2 renders limited multiplication of oncolytic herpes simplex virus-1 in human tumor cells

**DOI:** 10.1038/s41598-021-00691-y

**Published:** 2021-10-27

**Authors:** Yong Ren, Meiling Chen, Guangxian Wu, Dongmei Ji, Grace Guoying Zhou, Pei-Gen Ren, Wenmin Fu

**Affiliations:** 1 Shenzhen International Institute for Biomedical Research, Longhua District, Shenzhen, 518116 Guangdong China; 2 Research Center for Reproduction and Health Development, Institute of Biomedicine and Biotechnology, Shenzhen Institutes of Advanced Technology, Chinese Academy of Sciences, Shenzhen, Guangdong China; 3 School of Basic Medical Sciences, Guangzhou Medical University, Guangzhou, 511436 Guangdong China; 4 Shanghai Cancer Center and Shanghai Medical College, Fudan University, Shanghai, China

**Keywords:** Antivirals, Tumour virus infections, Innate immunity

## Abstract

Increasing studies demonstrated that oncolytic activities of oHSV-1 are limited to the capacity of virus replicating in tumors. In order to potentiate the oHSV-1 oncolytic activity and expand the application of oHSV-1 treatment in multiple types of tumors, it is critical to explore the potential factors or mechanisms mediating tumor resistance to oHSV-1 infection. Here we evaluated the levels of oHSV-1 multiplication in various tumor cell lines and showed that glioblastoma cell line A172 had the lowest virus yields but intrinsically accumulated the highest levels of Mx2 protein. Subsequently we demonstrated that genetic depletion of Mx2 specifically enhanced oHSV-1 productive replication in A172 cells through promoting the nuclear translocation of uncoated viral genomic DNA and down-regulating innate antiviral response. In the further investigation, we found that Mx2 knockdown could alter the intrinsic mRNA accumulation of diverse sets innate immune genes in A172 cells, in particular DHX36 and MyD88. Mx2 depletion led to a decrease in mRNA levels of MyD88 and DHX36 in A172 cells and MyD88/DHX36 knockdown increased virus yield in A172 cells and decreased the production of IFNα, activation of IRF3 activity and NF-κB signaling in A172 cells. This shed new lights on understanding the roles of some intrinsic antiviral genes in oHSV-1 resistance, facilitating to offer potential targets to improve oHSV-1 oncolytic efficacy and develop candidates of biomarkers to predict the efficiency of oHSV-1 multiplication in tumors.

## Introduction

Oncolytic virus is well known to selectively replicate in and lyse tumor cells, stimulating systemic innate/adaptive immune responses to achieve anti-tumor efficacy^1–3^. Oncolytic virotherapy using attenuated herpes simplex type-1 viruses (oHSV-1) has emerged as a hot alternative to radiotherapy and chemotherapy for various cancer treatments and been widely investigated in clinical trials. However, growing studies demonstrate that tumors showed varied responses to oHSV-1 treatment^4–6^. These might result from the differential capacity of oHSV-1 replicating in various tumor cells and the diverse ability of activating anti-tumor immunity. To implement the destruction of tumors, efficient intratumoral amplification of initial oHSV-1 inoculum in tumor cells is necessary and crucial. Deficient production of virus progeny diminishes the activation of immune responses and death of tumor cells. Hence, exploring the potential factors or mechanisms by which tumor cells exploit to resist to oHSV-1 replication is of great significance in developing and expanding the application of oHSV-1 anti-tumor therapy.

oHSV-1 as the foreign invader, can stimulate the antiviral response of the host immune system, even though the tumor is an immune-suppressive environment.

Therefore, intrinsic antiviral response or constitutive expression of antiviral genes could be the first obstacle for the efficient virus multiplication in some tumor cells.

Myxovirus resistance (Mx) proteins are one of the classical IFN-induced proteins and key players of the innate immune response to viral infection^7–12^. Humans usually express two paralogous *MX* genes (*MX1* and *MX2*)^13^. As one of them, human myxovirus resistance protein 2 (Mx2, also designated MxB) has been recently reported to be a pan-herpesvirus restriction factor, interfering with early steps of herpesvirus replication^7,14^. Mx2 blocks herpesvirus infection by inhibiting the delivery of incoming HSV-1 genomic DNA into the nucleus^7^. Additional mechanistic studies indicating that the N-terminal NLS, the formation of Mx2 dimers or oligomers, and GTP hydrolysis are important for the anti-herpesvirus activity of Mx2^9^. Besides, Schilling et al. found that IFNα failed to induce robust HSV-1 resistance in T98G glioblastoma cells lacking functional MX2 genes, demonstrating that Mx2 is a major mediator of herpesvirus resistance in IFN-treated cells^14^. We, therefore, evaluated the correlation of Mx2 with the tumor resistance to oHSV-1 replication in this study and investigated the mechanism by which Mx2 influences oHSV-1 productive replication and even oncolytic activity, to offer potential targets to improve oHSV-1 oncolytic efficacy and develop candidates of biomarkers to predict the efficiency of oHSV-1 multiplication in tumors.

## Results

### Differential capacity of oHSV-1 T1012G replicating in multiple human tumor cell lines

**Figure 1:**
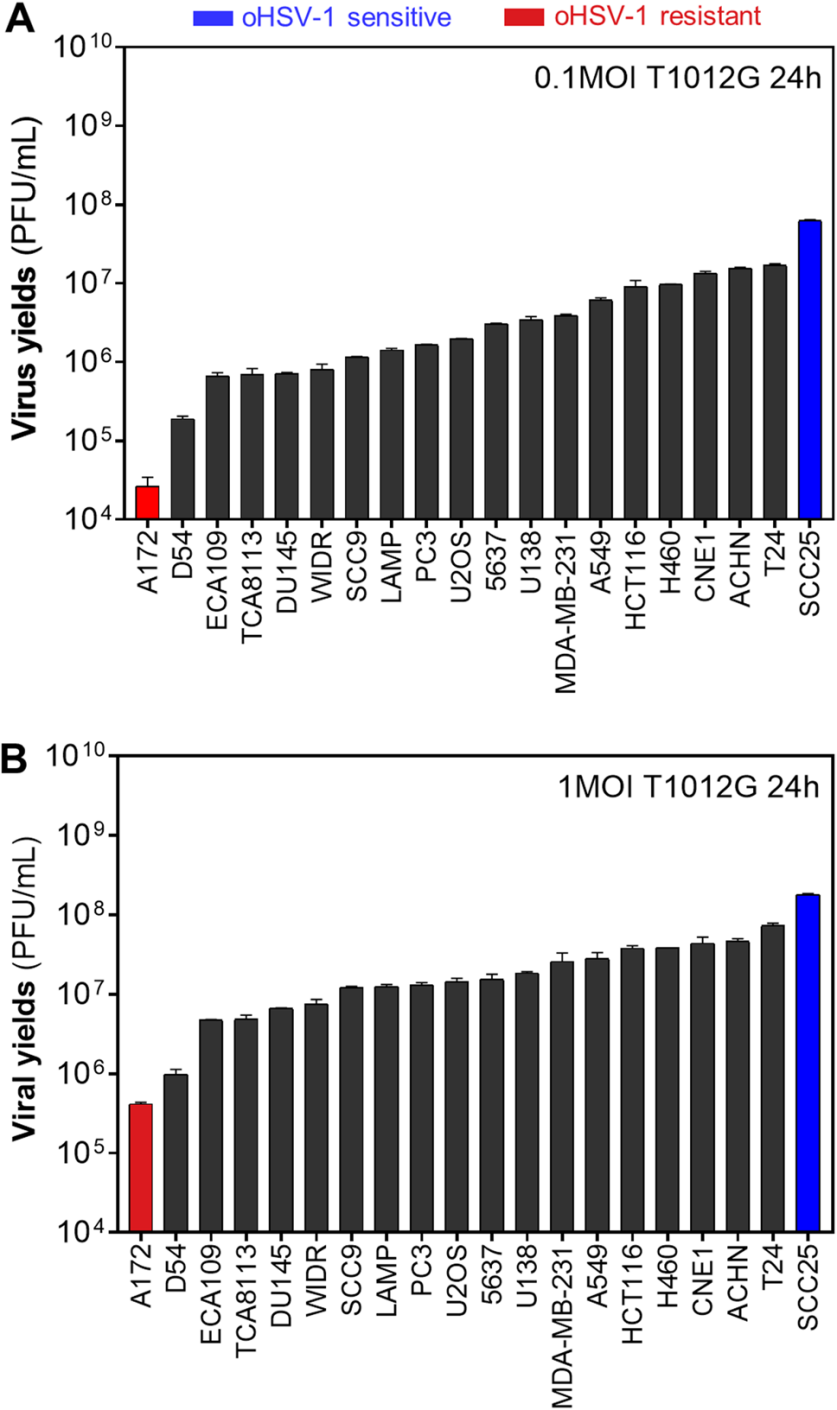
Differential capacity of oHSV-1 T1012G replicating in various human tumor cell lines. (**A**,**B**) Viral production of oHSV-1 T1012G in various human tumor cell lines. According to the capacity of viral proliferation, human glioblastoma cells A172 were defined as oHSV-1 resistant tumor cells (red marked), human tongue squamous carcinoma cell SCC25 were defined as oHSV-1 sensitive tumor cells (blue marked).

We evaluated 20 human tumor cell lines for their susceptibility to oHSV-1 T1012G infection. T1012G has been described elsewhere^15^ in which the inverted repeats of viral genome were replaced by sequences encoding the CMV promoter followed by three stop codons. T1012G is an attenuated HSV-1 without foreign genes inserted which was used as the backbone to develop new generations of oHSV-1. We infected each cell line with T1012G at a multiplicity of infection (MOI) of 0.1 and 1 infectious virus particles per cell. Virus-containing cells were harvested at 24 h post-infection to measure productive virus yields via the conventional plaque assay. The amounts of virus released by T1012G-infected cells greatly varied among 20 human tumor cell lines as shown in Fig. 1A,B, human tongue squamous carcinoma cell SCC25 (marked in blue) had the most virus yields and were highly permissive to T1012G viral lytic replication, while human glioblastoma cells A172 (marked in red) had limited virus multiplication and showed obvious resistance to T1012G productive infection. According to the differential efficiency of viral proliferation in these human tumor cell lines, A172 was defined as oHSV-1 resistant tumor cell line and used for further investigation of the resistance mechanism to oHSV-1 infection, SCC25 was oHSV-1 sensitive tumor cell lines and applied for comparison.

### Mx2 depletion enhanced oHSV-1 T1012G lytic replication in resistant A172 cells

**Figure 2:**
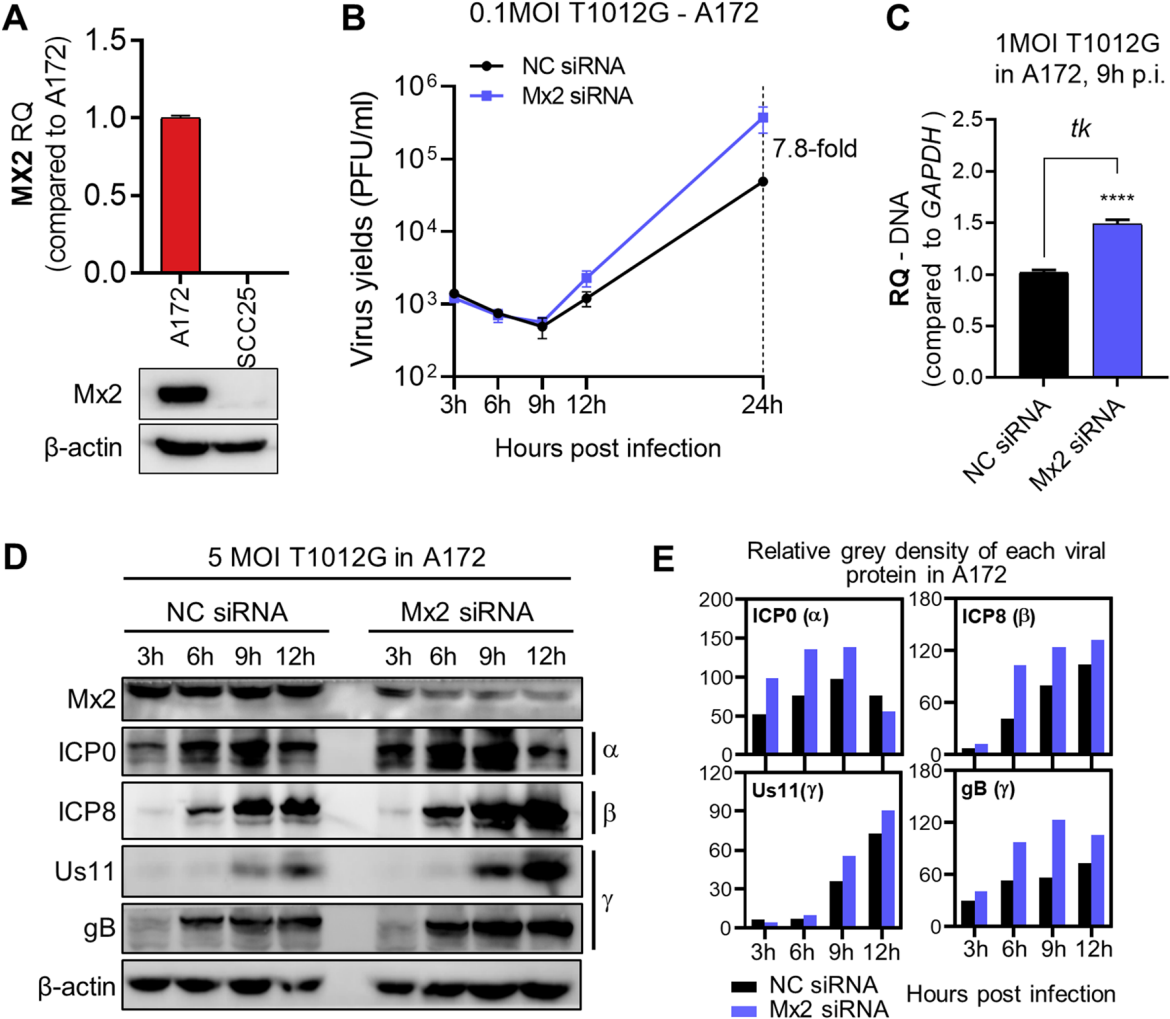
Depletion of Mx2 in resistant A172 cells enhanced oHSV-1 T1012G lytic replication. (**A**) mRNA accumulation of Mx2 in A172 and SCC25 cells. (**B**) Virus yields in Mx2-depleted A172 cells. Virus progeny were harvested at the indicated time and titrated in Vero cells. (**C**) Viral DNA copy numbers in Mx2-depleted A172 cells. Viral DNA was isolated and analyzed by the qPCR assay. Primers set of ICP0 and *tk* were used to detect DNA copies. RQ: relative quantity, normalized to GAPDH DNA. (**D**) Expression of viral proteins in Mx2-depleted A172 cells at indicated time points post 5 MOI T1012G infection. Blots were cut and probed with ICP0 (α); ICP8 (β); Us11, gB (γ) or β-actin antibodies, respectively. β-actin serves as a loading control. (**E**) The band density of viral protein was measured by the grey analysis. Relative density was normalized to β-actin.

Mx2 as a pan-herpesvirus restrictor interfering with viral replication^14^ was detected with high accumulation of intrinsic mRNA and protein in oHSV-1 resistant (A172) cells when compared to oHSV-1 sensitive (SCC25) cells (Fig. 2A). Therefore, we hypothesized that abundant Mx2 accumulation limits the ability of oHSV-1 T1012G replicating in A172 cells. In order to verify this, we transfected Mx2 small interfering RNA (siRNA) to knock down the cellular expression of Mx2 in A172 cells. The impact of Mx2 depletion on T1012G virus yields at indicated time points were investigated using the plaque assay. oHSV-1 virus titer in Mx2-depleted A172 cells was increased about eightfold compared to those in mock-depleted A172 cells at 24 h, the late step of viral lytic replication (Fig. 2B).

We also investigated viral DNA copy numbers and viral protein expression to determine if Mx2 depletion influenced T1012G lytic replication. As shown in Fig. 2C, Mx2 knockdown led to a significant increase of ICP0 DNA and *tk* DNA in resistant cell line A172. Western blot was applied to determine the expression levels of indicated viral proteins (immediate early-α, early-β, late-γ) (Fig. 2D,E). The results showed overall increased protein expression of ICP0 (α), ICP8 (β), Us11 (γ) and gB (γ) during viral productive infection.

Taken together, these results showed Mx2 depletion promoted T1012G lytic replication in A172 cells, suggesting that Mx2 may act as a restrictive factor to regulate the lytic replication of oHSV-1 T1012G in resistant cell line A172.

### Mx2 depletion improved the nuclear translocation of oHSV-1 viral genome in resistant cell line A172

**Figure 3:**
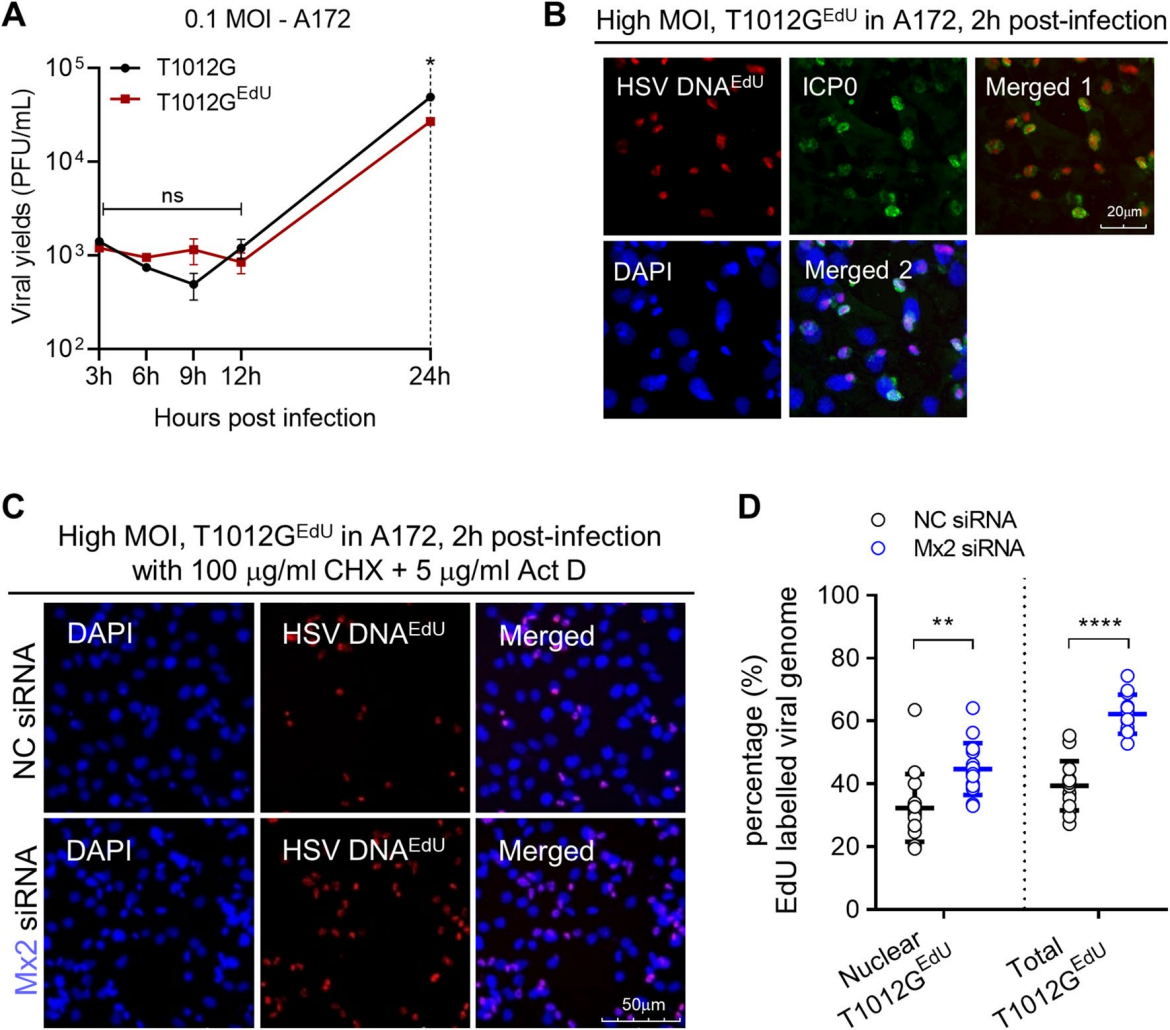
Effects of Mx2 depletion on nuclear translocation of oHSV-1 viral genome in A172 cells. (**A**) The growth curve of EdU-labeled oHSV-1 T1012G (T1012G^EdU^) from A172 cells. Virus progeny were harvested at the indicated time and titrated in Vero cells. (**B**) The infection of T1012G^EdU^ in A172 cells was assessed by immunofluorescence assay. Viral genomic DNA^EdU^ (red), ICP0 (green), DAPI (blue) as visualized. (**C**) Nuclear import of EdU-labeled viral genomic DNA in Mx2-depleted A172 cells. Mx2-depleted or mock-depleted A172 cells were infected with high MOI of EdU-labeled T1012G virus for 2 h. Viral genomic DNA^EdU^ (red), ICP0 (green), DAPI (blue) were immune-stained as indicated. Samples were analyzed with the microscope at magnification, ×20. Scale bar, 50 mm. (**D**) Percentage of EdU labeled viral genomes coinciding with the nuclear mask. 15 fields were randomly chosen from the samples. Numbers of cell and EdU-labeled dot were counted in merged (DNA^EdU^ + DAPI) images. Percentage = numbers of EdU dots (nuclear or total)/numbers of cell in each field. (**p < 0.01; compared with NC siRNA-treated A172 cells. Error bars represent SD).

Although we have shown that Mx2 correlates with limited oHSV-1 multiplication in A172 cells, it is important to explore the mechanism of how Mx2 affects viral infection. Mx2 has been reported that could block herpesvirus infection by interfering with the translocation of genomic viral DNA into the nucleus^7,14,16,17^. Therefore, we hypothesized that Mx2 inhibits the delivery of incoming viral DNA to the nucleus for repressing oHSV-1 T1012G productive infection in resistant cell line A712 and that knockdown of Mx2 would enhance nuclear translocation of viral genome in A172 cells. To investigate the hypothesis, we generated EdU(5-ethynyl-2′-deoxyuridine)-genome labeled oHSV-1 T1012G (T1012G^EdU^), which could make uncoated and condensed viral genome DNA detectable using combined immunofluorescence and click chemistry approach^18,19^. The generation of T1012G^EdU^ was described in “Material and methods”. Firstly, we investigated the growth curve of T1012G and T1012G^EdU^ in resistant cell line A172. As shown in Fig. 3A, at immediate-early and early stages of viral lytic replication, no obvious difference of virulence between T1012G and T1012G^EdU^ was observed. T1012G presents slight increases in virus yields at 24 h post-infection when compare to T1012G^EdU^. After HSV-1 particle entry into the cytoplasm, viral genome DNA would be uncoated from the capsid and translocated to the nucleus at a very early time^20,21^, detected at 30 min post-infection and peaked at 120 min post-infection^8^. Then, we infected A172 cells with T1012G^EdU^ at high MOI (MOI = 15) for 2 h, followed with the immunofluorescence assay to determine T1012G^EdU^ infection in resistant cell line A172 and the cellular location of viral DNA and ICP0 protein. At 2 h post-infection, EdU-labeled viral genome (red) was present in ICP0 (green)-expressing cells (Fig. 3B).

Based on these observations, we next used to investigate the nuclear translocation of incoming viral DNA via combined immunofluorescence and click chemistry approach. The resistant cell line A172 was transfected with Mx2 siRNA or NC siRNA. 24 h post-transfection, Mx2-depleted or mock-depleted A172 cells were exposed to high MOI (MOI = 15) EdU genome-labeled T1012G^EdU^ in the presence of actinomycin D (Act D) and cycloheximide (CHX). The addition of Act D and CHX was to minimize the consumption of incoming viral DNA, blocking the viral mRNA transcription and protein synthesis. Both reagents have previously shown not to interfere with HSV-1 genome uncoating and nuclear translocation^22^. At 2 h post-infection, cells were progressed for the immunofluorescence assay. Viral genomes (red) were visualized using the Click-iT Plus EdU imaging kit, Alexa Fluor 647 according to the manufacturer’s instructions. The nuclei were stained with DAPI (blue). Mx2-depleted A172 cells exhibited more EdU-labeled viral genomic DNA when compared to mock-depleted A172 cells (Fig. 3C). Moreover, number counting was performed to quantify the nuclear import of uncoated viral genomes in 15 randomly chosen fields of each slide by using Image J software. Percentages of EdU labeled viral genomes coinciding with the nuclear were calculated and plotted as Fig. 3D. Percentage = numbers of EdU dots (nuclear or total)/numbers of cells in each field. A172 cells with silenced Mx2 expression exhibited a 1.4-fold increase of viral genomes translocated to the nucleus compared to mock-depleted A172 cells. This suggests that Mx2 repressed the nuclear translocation of viral genomes in the resistant cell line A172. Besides, a 1.3-fold increase of total EdU labeled uncoated viral genomes were observed in Mx2-depleted A172 cells. This may indicate that the depletion of Mx2 possibly facilitated the uncoating of viral genomes from capsid.

### Mx2 knockdown alters antiviral innate response and intrinsic mRNA accumulation of innate immune genes in the resistant cell line A172

**Figure 4:**
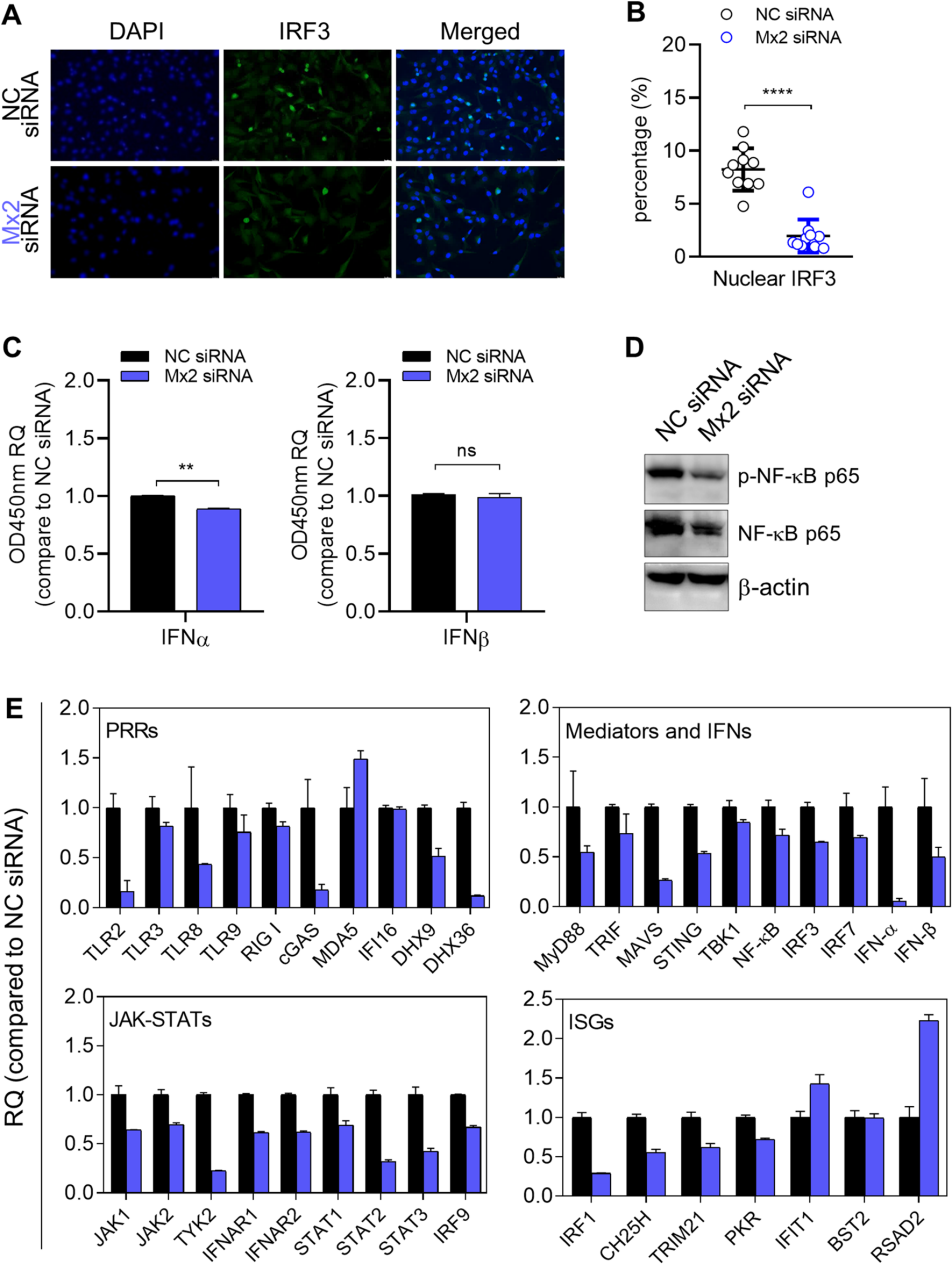
The effects of Mx2 depletion on antiviral innate response and intrinsic mRNA accumulation of innate immune genes in resistant A172 cells. (**A**) Investigation of nuclear IRF3 in A172 cells via immunofluorescence staining. (**B**) Quantification of IRF3 localized in the A172 nucleus by automatic counting using Image J software. 10–15 fields of each slide were randomly chosen for counting the numbers of IRF3-DAPI coincident dots. (**C**) ELISA quantification of secreted IFNα and IFNβ in cell supernatant. RQ: relative quantity, values that correspond to optical densities at OD 450 nm compared to values in NC siRNA-transfected A172 cells. (*p < 0.05; **p < 0.01; compared with NC siRNA-treated A172 cells. Error bars represent SD.) (**D**) Expression of effector proteins associated with the NF-κB signaling pathway. Blots were cut and probed with indicated antibodies, respectively. β-actin serves as a loading control. (**E**) mRNA accumulation of indicated immune genes in Mx2-depleted A172 cells. Mx2-depleted or mock-depleted A172 cells were harvested at 24 h post-transfection. Total RNA was isolated and analyzed by qPCR assay for mRNA levels of indicated immune genes. Used primers are shown in Table 3. RQ: relative quantity, compared to transcript levels in mock-depleted A172 cells. Bar, mean ± SD. GAPDH serves as a normalization control.

As we shown above, Mx2 directly interferes with oHSV-1 T1012G lytic replication in the resistant cell line A172 through its own function. Besides, we also investigated the effect of Mx2 depletion on basal antiviral response in A172 cells, like the activation of IRF3/NF-κB signaling and interferon production. Firstly, immunofluorescence assay was applied to examine IRF3 nuclear localization in A172 cells when Mx2 was depleted (Fig. 4A). Number counting was performed to quantify the nuclear import of uncoated viral genomes in 10–15 randomly chosen fields of each slide by using Image J software. Percentages of IRF3 coinciding with the nuclear were calculated and plotted as Fig. 4B. The percentage of nuclear IRF3 was significantly reduced in Mx2-depleted A172 cells, compared to the mock-depleted A172 cells. Secondly, the levels of secreted IFNα and IFNβ in cell supernatant were detected and only significant decreases of the secreted IFNα were generally observed in Mx2-depleted A172 cells. The levels of IFNβ in the cell supernatant showed no change (Fig. 4C). Thirdly, similar experiments were performed to examine the expression of the effector associated with the NF-κB signaling pathway. Western blot analysis in Fig. 4D showed that Mx2 knockdown led to a notable reduction in the levels of total p65 and phosphorylated-p65 in A172 cells.

Additionally, we examined the mRNA accumulation of many innate immune genes in Mx2-depleted A172 cells via qPCR assay to explore the potential cell-intrinsic innate immune factors contributing to the activity of Mx2 against oHSV-1 replication in the resistant cell line A172. Tested innate immune genes were grouped into 4 according to their molecular functions: pattern recognition receptors (PRRs), mediators and IFNs, JAK-STATs, and ISGs. As shown in Fig. 4E, these innate immune genes exhibited differential responses to Mx2 knockdown in A172 cells. A chart with fold change and the *p*-value was made for a better comparison of immune gene expression between Mx2-depleted and mock-depleted A172 cells (Table 1). In the group of PRRs, *TLR2*, *cGAS*, *DHX9*, and *DHX36* showed a 50% decrease in mRNA accumulation when Mx2 was depleted in A172 cells. *MyD88*, *MAVS*, *STING*, *IFNα*, and *IFNβ* in the mediators and IFNs group present approximately 50% decrease of mRNA levels in Mx2-depleted A172 cells. Besides, Mx2 knockdown also caused significantly reduced mRNA accumulation of *TYK2*, *STAT2*, *STAT3*, *IRF1*, and *BST2* in the resistant cell line A172. Only *RSAD2* was found a conspicuous increase in gene expression in Mx2-depleted A172 cells.

**Table 1:** Summarized comparison of immune gene expression between Mx2 siRNA and NC siRNA-transfected A172 cells with fold change and *p*-value.

PRRS			Mediators and IFNs		
Gene	Fold change	*p*-value	Gene	Fold change	*p*-value
*TLR2*	↓	**	*MyD88*	↓	*
*TLR3*	–	–	*TRIF*	–	–
*TLR8*	–	–	*MAVS*	↓	***
*TLR9*	–	–	*STING*	↓	***
*RIG I*	–	–	*TBK1*	–	–
*cGAS*	↓	–	*NF-κB*	–	–
*MDA5*	–	–	*IRF3*	–	–
*IFI16*	–	–	*IRF7*	–	–
*DHX9*	↓	**	*IFNα*	↓	***
*DHX36*	↓	***	*IFNβ*	↓	*

JAK-STATs			ISGs		
Gene	Fold change	*p*-value	Gene	Fold change	*p*-value
*JAK1*	–	–	*IRF1*	**↓**	***
*JAK2*	–	–	*CH25H*	–	–
*TYK2*	**↓**	***	*TRIM21*	–	–
*IFNAR1*	–	–	*PKR*	–	–
*IFNAR2*	–	–	*IFIT1*	–	–
*IFNGR1*	–	–	*BST2*	**↓**	ns
*IFNGR2*	–	–	*RSAD2*	↑	***
*STAT1*	–	–			
*STAT2*	**↓**	***			
*STAT3*	**↓**	***			
*IRF9*	–	–			

↑ Fold change ≥ 2 in Mx2-depleted A172 cells.

↓ Fold change ≤ − 2 in Mx2-depleted A172 cells.

– represents − 2 < Fold change < 2.

ns, not significant.

*p < 0.05; **p < 0.01; ***p < 0.001.

These results indicated that depletion of Mx2 could regulate oHSV-1 T1012G productive replication in the resistant cell line A172 through mediating antiviral innate response.

### Identification of immune factors responsible for increased virus production in the resistant cell line A172 mediated by Mx2 depletion

As described above, differential mRNA accumulation of innate immune genes were observed in Mx2-depleted A172 cells. According to their molecular functions and interactive relationship, we summarized the regulatory network among these significantly altered innate immune genes as shown in Table 1. Among them, the following 4 regulatory axes potentially associated with Mx2 mediated oHSV-1 resistance in A172 cells were sorted out: ① cGAS → STING, ② MAVS → STING, ③ TLR2 → MyD88, ④ DHX9/36 → MyD88. Subsequently, a series of experiments were carried out to explore the interrelation.

**Figure 5:**
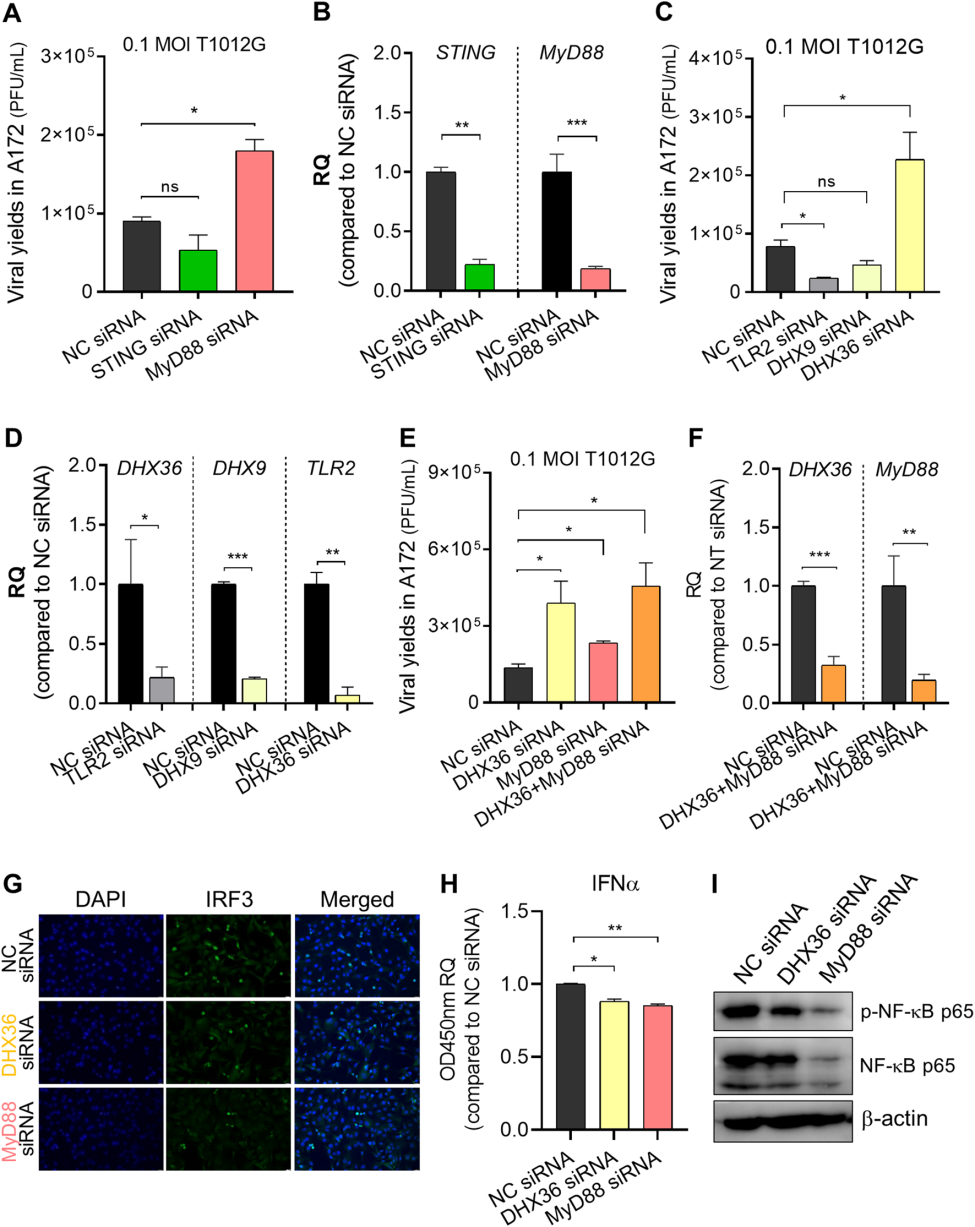
Identification of immune factors responsible for increased virus production in the resistant cell line A172 mediated by Mx2 depletion. (**A**–**F**) A172 cells were transfected with indicated siRNAs or NC siRNA, followed by exposure to 0.1 MOI oHSV-1 T1012G for 2 h at 24 h post-transfection. Virus progeny were harvested at 24 h post-infection and titered in Vero cells. (**A**) Viral yields in MyD88 or STING-depleted A172 cells; (**C**) Viral yields in DHX36, DHX9 or TLR2-depleted A172 cells; (**E**) Viral yields in MyD88, DHX36 or both-depleted A172 cells; (**B**,**D**,**F**) Knockdown efficiency of MyD88, STING, DHX36, DHX9 and TLR2 siRNA were analyzed by the qPCR assay. RQ: relative quantity, compared to transcript levels in NC siRNA-transfected A172 cells. Error bars represent SD. GAPDH serves as a normalization control. A172 cells were depleted of DHX36, MyD88 or mock-depleted by siRNA transfection, 24 h later, cells were harvested for the following indicated tests. (**G**) Investigation of nuclear IRF3 in A172 cells via immunofluorescence staining. (**H**) ELISA quantification of secreted IFNα in cell supernatant. RQ: relative quantity, values that correspond to optical densities at OD 450 nm compared to values in NC siRNA-transfected A172 cells. (*p < 0.05; **p < 0.01; compared with NC siRNA-treated A172 cells. Error bars represent SD.) (**I**) Expression of effector proteins associated with the NF-κB signaling pathway. Blots were cut and probed with indicated antibodies, respectively. β-actin serves as a loading control.

Firstly, we investigated the potential roles of STING and MyD88 which act as nodes of the above regulatory paths in affecting oHSV-1 infection in the resistant cell line A172. STING or MyD88 was depleted in A172 cells via siRNA transfection. The production of oHSV-1 T1012G infectious particles in STING- or MyD88-depleted A172 cells was examined by the plaque assay. As shown in Fig. 5A, MyD88-depleted A172 cells exhibited a twofold increase of virus yields compared to mock-depleted A172 cells, while no obvious change was observed in STING-depleted A172 cells. Meanwhile, an RT-qPCR assay was performed to identify the knockdown efficiency of MyD88 and STING siRNAs (Fig. 5B). These results suggest that MyD88 could be the immune effector, induced by Mx2 for limiting oHSV-1 T1012G virus yields in the resistant cell line A172.

Next, we performed similar experiments to investigate the upstream effector in the regulatory axes of MyD88 (③ TLR2 → MyD88, ④ DHX9/36 → MyD88). DHX36 depletion enhanced viral production in A172 cells, DHX9 knockdown showed no effect on virus yields. In contrast, loss of TLR2 in A172 cells decreased the release of infectious virus particles (Fig. 5C). Likewise, RT-qPCR assays were performed to examine the knockdown efficiency of DHX36, DHX9, and TLR2. siRNA transfection led to a significant decrease in mRNA accumulation of DHX36, DHX9, and TLR2 (Fig. 5D). As our results showed, DHX36 and MyD88 would be the potential immune effector contributing to the activity of Mx2 against oHSV-1 infection in the resistant cell line A172. Further, we determined double knockdown of MyD88 and DHX36 increased oHSV-1 virus yields in the resistant cell line A172 (Fig. 5E). The patterns of MyD88-depleted and DHX36-depleted were identical to those described above. And the knockdown efficiency was determined via qRT-PCR. Co-transfection of MyD88 and DHX36 siRNA led to notable decreases of MyD88 and DHX36 mRNA levels in A172 cells (Fig. 5F).

In summary, our results showed that Mx2 knockdown negatively regulates DHX36 and MyD88 expression in the resistant cell line A172, and the depletion of DHX36 and MyD88 increases oHSV-1 virus yields in A172 cells, suggesting DHX36 and MyD88 might be restrictive factors mediated by Mx2 for oHSV-1 multiplication in the resistant cell line A172.

### The effects of DHX36 and MyD88 knockdown on the basal innate immune response in the resistant cell line A172

Additionally, we investigated the effect of depletion of DHX36, or MyD88 on the activation of IRF3/NF-κB signaling and interferon production in the resistant cell line A172. Firstly, an immunofluorescence assay was applied to investigate IRF3 nuclear localization in A172 cells when DHX36, or MyD88 were respectively depleted (Fig. 5G). The percentage of nuclear IRF3 was notably reduced when DHX36 or MyD88 was depleted in A172 cells.

Besides, the levels of secreted IFNα and IFNβ in cell supernatant were detected and only significant decreases of the secreted IFNα were generally observed in DHX36-depleted, and MyD88-depleted A172 cells (Fig. 5H).

Additionally, similar experiments were performed to examine the expression of the effector associated with the NF-κB signaling pathway. Western blot analysis in Fig. 5I showed that the levels of total p65 and phosphorylated-p65 were notably reduced in the resistant cell line A172 when either DHX36, or MyD88 was depleted.

Collectively, our results indicate a regulatory axis of DHX36 and MyD88 mediated by Mx2 affects oHSV-1 lytic replication in the resistant cell line A172 possibly through regulating the production of IFNα and the activation of IRF3/NF-κB signaling pathway.

### The potential association of Mx2 with oHSV-1 resistance in other human tumor cell lines

**Figure 6:**
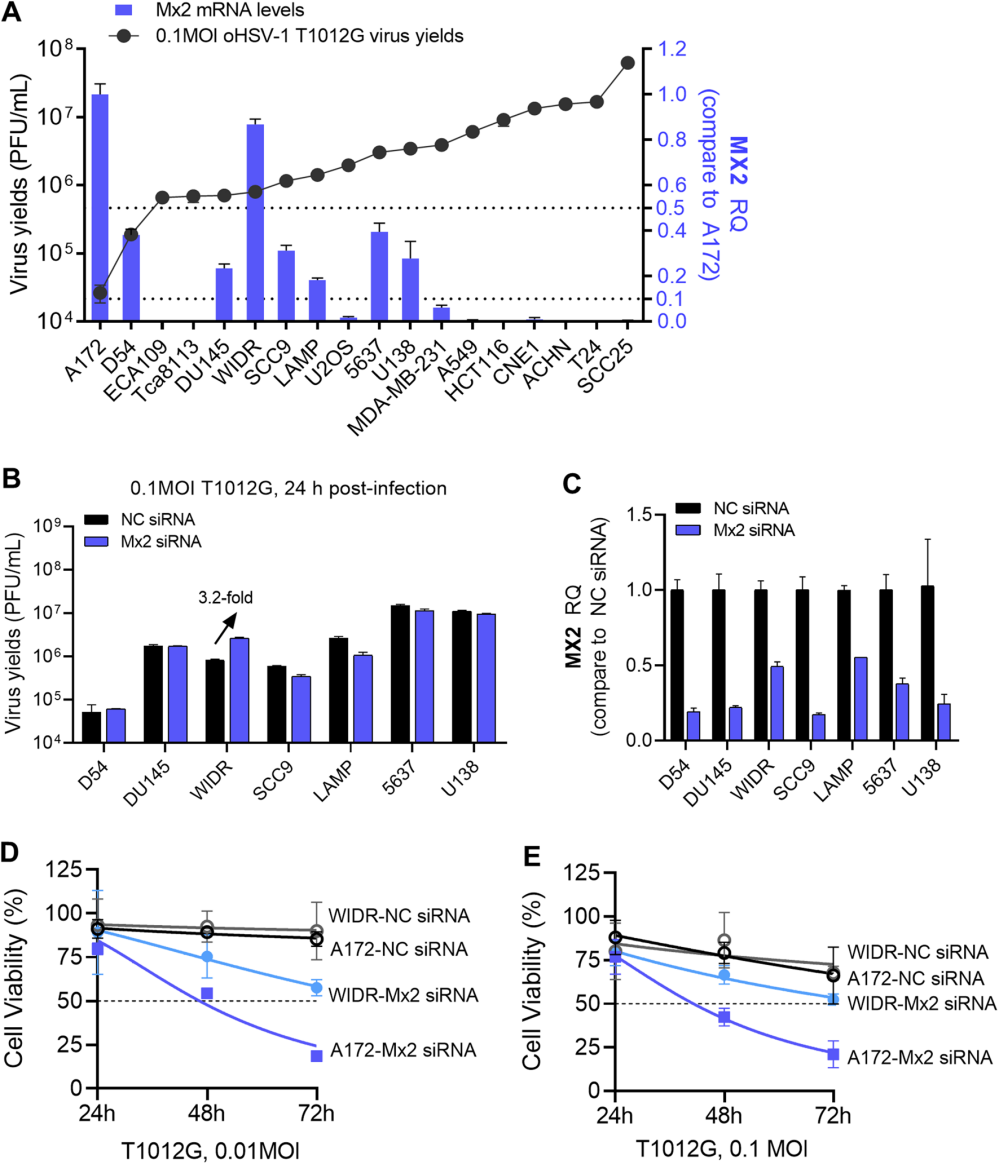
The potential association of Mx2 with oHSV-1 resistance in other human tumor cell lines. (**A**) The mRNA accumulation of Mx2 in other human tumor cell lines. RQ: relative quantity, values were compared with Mx2 mRNA levels in A172 cells. (**B**) Virus yields in Mx2-depleted D54, DU145, WIDR, SCC9, LAMP, 5637 and U138. Mx2 was knockdown in above human tumor cells lines by siRNA transfection. Treated cells were exposed to 0.1 MOI T1012G, virus progeny was harvested at the 24 h post-infection and titrated in Vero cells. (**C**) Investigation of Mx2 knockdown efficiency by RT-qPCR assay. (**D**,**E**) The cell viability of Mx2-depleted A172 cells and WIDR cells. Mx2-depleted and mock-depleted A172 cells or WIDR cells were infected with oHSV-1 T1012G at an MOI of 0.01, 0.1 MOI, and cell viability was analyzed every 24 h over a time course of 72 h with CCK-8 Assays Kit.

We have demonstrated the roles of Mx2 plays in regulating oHSV-1 lytic replication in resistant cell line A172. Here we wonder whether Mx2 is a general restrictive factor of oHSV-1 virus multiplication in resistant tumor cell lines, possibly be taken as a candidate of biomarkers to predict the efficiency of oHSV-1 replication in tumors and oncolytic activity. The intrinsic mRNA accumulation of Mx2 in other human tumor cell lines listed in Table 2 was examined by RT-qPCR assay. As shown in Fig. 6A, to some extent, Mx2 accumulation associates with the capacity of oHSV-1 T1012G replicating in these tumor cell lines. Relative higher mRNA levels of Mx2 were observed in the relative resistant cell lines. Next, we explored the possible effects of Mx2 depletion on those tumor cell lines in which the Mx2 mRNA levels are higher than 10% of that in A172 cells. D54, DU145, WIDR, SCC9, LAMP, 5637, and U138 cells were depleted of Mx2 or mock-depleted by siRNA transfection, following infected with 0.1 MOI oHSV-1 T1012G at 24 h post-transfection. Virus-containing cells were harvested at 24 h post-infection to measure productive virus yields via the plaque assay. Only Mx2 depletion in WIDR cells resulted in a 3.2-fold increase in virus yields. No significant change was observed in other tumor cells (Fig. 6B). Mx2 knockdown efficiency in indicated tumor cells were investigated by RT-qPCR (Fig. 6C).

**Table 2:** Cell lines used in this study.

Cell line	Name	Culture medium	Source
5637	Human bladder carcinoma cell	1640 + 10%FBS	ATCC
A172	Human glioblastoma cell	DMEM + 10%FBS	ATCC
A549	Human lung carcinoma cell	DMEM + 10%FBS	Gift from Sun Yat-sen University
ACHN	Human renal cell adenocarcinoma cell	DMEM + 10%FBS	ATCC
CNE1	Human nasopharyngeal carcinoma cell	1640 + 10%FBS	Gift from Sun Yat-sen University
D54	Human glioblastoma cell	DMEM + 10%FBS	ATCC
DU145	Human prostate carcinoma cell	1640 + 10%FBS	ATCC
ECA109	Human esophageal carcinoma cell	1640 + 10%FBS	Gift from Sun Yat-sen University
HCT116	Human colorectal carcinoma cell	DMEM + 10%FBS	Gift from Sun Yat-sen University
LAMP	Human lung adenocarcinoma cell	1640 + 10%FBS	Gift from Guangzhou Medical University
MDA-MB-231	Human breast cancer cell	DMEM + 10%FBS	Gift from Sun Yat-sen University
PC3	Human prostate carcinoma cell	1640 + 10%FBS	Gift from Shanghai Jiaotong University
SCC9	Human tongue squamous carcinoma cell	DMEM + 10%FBS	ATCC
SCC25	Human tongue squamous carcinoma cell	DMEM + 10%FBS	ATCC
Tca8113	Human tongue squamous carcinoma cell	1640 + 10%FBS	ATCC
U138	Human glioblastoma cell	DMEM + 10%FBS	ATCC
U2OS	Human bone osteosarcoma cell	1640 + 10%FBS	Gift from University of Chicago
Vero (CCL-81)	African green monkey kidney cell	DMEM + 5%FBS	ATCC

As we have shown above that Mx2 depletion enhanced oHSV-1 T1012G replication in A172 and WIDR cells, here we further investigated whether Mx2 depletion could promote virus-induced loss of A172 and WIDR cells viability. A172 and WIDR cells were depleted of Mx2 or mock-depleted by siRNA transfection, 24 h post-transfection, following infected with oHSV-1 T1012G at an MOI of 0.01, 0.1MOI. The cell viability was analyzed every 24 h over a time course of 72 h with CCK-8 Assays Kit. Mx2-depleted A172 cells displayed a significant drop in viability (≥ 50%) starting from 48 h post-infection at an MOI of 0.01 and 0.1 (Fig. 6D,E), a 50% loss of viability was found in Mx2-depleted WIDR cells at 72 h post-infection. These results suggest that Mx2 knockdown renders A172 and WIDR cells more susceptible to oHSV-1 induced oncolysis.

All these above showed that silenced Mx2 expression in A172 and WIDR cells improved oHSV-1 virus multiplication, and a high abundance of Mx2 mRNA was found both in A172 and WIDR cells. These indicate that Mx2 may not be a universal factor responsible for the resistance of tumor cells to oHSV-1 infection in a broad sense, while there might be a certain threshold of Mx2 accumulation. Intrinsic Mx2 levels in some tumor cells that reach the threshold will render resistance or semi-permissivity to oHSV-1 infection. More studies need to be performed in the future to identify if Mx2 could be a potential biomarker.

## Discussion

In this study, we showed the differential capacity of oHSV-1 replicating in various human tumor cells. And it is well-known that efficient intratumoral amplification of initial oHSV-1 inoculum in tumor cells is necessary and crucial for the effective destruction of tumors. Deficient production of virus progeny diminishes the activation of immune responses and the death of tumor cells. Hence, exploring the potential factors or mechanisms by which tumor cells exploit resistance to oHSV-1 replication is of great significance in developing and expanding the application of oHSV-1 anti-tumor therapy.

Human myxovirus resistance protein 2 (Mx2), which has been demonstrated its inhibitory roles in affecting herpesvirus infection of all three subfamilies^7,14,23^, were found highly expressed in some resistant cell lines in this report. Moreover, genetic depletion of Mx2 enhanced oHSV-1 lytic replication in A172 cells, but not in D54 and DU145 cells, suggesting that the association of Mx2 with restricted oHSV-1 infection is cell-specific, not universal. Although A172, D54, and DU145 are described as human glioma cell lines, they have different mutations or overexpression of some genes, and even various tumorigenicity, which may be the explaination for the different roles of Mx2 plays in these 3 glioblastoma cell lines. Interestingly, Mx2 depletion in WIDR cells improved oHSV-1 virus multiplication. And we found that A172 and WIDR cells exhibited similar and abundant Mx2 accumulation. These generated a hypothesis that there might be a certain threshold of Mx2 accumulation. Intrinsic Mx2 levels in some tumor cells that reach the threshold will render resistance or semi-permissivity to oHSV-1 infection. More exact mechanisms remain to be explored.

Besides, we explored the mechanism of how Mx2 affects viral lytic replication. Mx2 depletion was found that promoted the delivery of uncoated viral genomic DNA to the nucleus in the resistant cell line A172. Viral replication initiates only after the viral genome is delivered to the nucleus, where mRNA transcription and DNA replication occurred to propagate infection^24–26^. Therefore, significant increases in the nuclear oHSV genomic DNA template are likely to play a role in enhanced viral lytic replication. Furthermore, knockdown of Mx2 in A172 cells was found to delay the progression of cell cycle to the G2 phase and arrest numerous cells at the G1/S phase (Figure S1). HSV-1 has been found able to address various cellular pathways including cell cycle to facilitate its replication and spread. HSV-1 infection induces an arrest of the cell cycle at the G1/S phase to create an optimized cellular environment that is conducive to viral replication^27,28^. Mx2-mediated disturbed distributions of cell-cycle interfere with oHSV-1 replication in the resistant cell line A172. These results indicate that Mx2 regulates oHSV-1 infection not only directly through affecting the virus life-cycle, but also indirectly through interfering with the environment provided by tumor cells.

Additionally, we showed a multiple-way resistance mechanism that originated from Mx2 exists in the resistant cell line A172. In this report, we found that Mx2-mediated down-regulation of DHX36 and MyD88 likely contribute to restricted oHSV-1 multiplication, since we observed that depletion of DHX36 and MyD88 could enhance virion production in A172 cells, meanwhile IFNα production and IRF3/NF-κB activation were moderately increased when Mx2, DHX36, or MyD88 were separately depleted in A172 cells. These indicate that Mx2-mediated ISGs expression or interferon production could repress oHSV-1 infection in the resistant cell line A172.

This is the first time to demonstrate that Mx2 can also indirectly affect HSV-1 infection via interfering with the intrinsic expression of immune effector in tumor cells. Mx2 has been disclosed its potential activities against herpesviruses until 2018^7,9,10,14^, although it has been long known as a potent, IFN-induced inhibitor of HIV-1 infection^8,9,16,29,30^. The mechanisms by which Mx2 affects viral infection mainly rely on the guanosine triphosphatases (GTPases) domain, which is associated with targeting the viral capsid and hindering uncoating/nuclear accumulation of viral genome DNA^17,29,30^. While the impact of Mx2 expression on cellular immune response against viral infection is barely mentioned. In this report, we sought to understand the comprehensive roles of Mx2 in tumor resistance to oHSV-1 infection, evaluating whether Mx2 depletion could reduce constitutive antiviral response to promote viral propagation in the resistant tumor cells. Although Mx2 depletion only led to eightfold increases in oHSV-1 T1012G virus yields in A172 cells which were still way less than that in the sensitive cell line SCC25. This indicates that limited virus multiplication of oHSV-1 in resistant cell line A172 is caused by multiple factors, Mx2 is one of the effector involved.

Understanding of the intrinsic antiviral genes in the resistant tumors and their roles in oHSV-1 resistance is aimed to develop candidates of biomarkers to predict the efficiency of oHSV-1 multiplication in tumors or offer potential targets to improve oncolytic efficacy. However, the immune response generated by the oncolytic virus has two-sided functions. Suppressed antiviral response facilitates active viral infection and induced tumor cell lysis, while it would weaken the immune response against the tumor cells. Therefore, it is crucial to balance the immune response between defending against viral infection and targeting tumors. These questions remain to be fully addressed by further investigations.

## Materials and methods

### Cells

All cells listed in Table 2 were cultured in the indicated medium, supplemented with indicated serum, 100 IU/ml penicillin and 100 μg/ml streptomycin (Invitrogen) at 37 °C, 5% CO_2_.

### Viruses

HSV-1 recombinant virus T1012G was previously described^15^. EdU genome-labeled T1012G (T1012G^EdU^) was generated as follows. Vero(CCL-81) cells were infected with 0.1 MOI T1012G and incubated until a complete cytopathic effect developed. EdU was added to the cells at 2 h and 24 h post-infection to a final concentration of 10 μM. EdU was from Click-iT Plus EdU imaging kit purchased from Invitrogen. The cell medium was harvested at approximately 72 h post-infection, and cell debris was removed by low-speed centrifugation. The virus-containing cells were collected, aliquoted and stored at − 80 °C for further titration analysis.

### Virus titration

Virus-containing cells were harvested and lysed with three freeze-and-thaw cycles. The virus titer was measured by conventional plaque assay. Vero(CCL-81) cells seeded on T25 flask were overlaid with serial tenfold-diluted virus suspensions in a duplicate manner and incubated at 37 °C, 5% CO_2_ for 1 h. After virus adsorption, suspensions were aspirated, cells were washed twice with PBS and incubated with fresh medium for 3 days to allow plaque formation. Cells were fixed and stained with crystal violet solution for plaque counting. Virus titer was calculated from the number of formed plaques per milliliter of sample and expressed as PFU/ml.

### Immunoblot assay and antibodies

Cells were harvested at indicated time points and lysed with RIPA lysis buffer (Beyotime) supplemented with 1 mM protease inhibitor PMSF (Beyotime). Protein lysates were separated on SDS-containing polyacrylamide gels, blotted on PVDF membranes and probed with appropriate antibodies. The following antibodies were used in this study: Mx2 rabbit monoclonal antibody (CST, #E7Y8H), HSV-1 ICP8 antibody (Abcam, ab20194), other viral proteins ICP0, ICP27, gB, ICP22, and Us11 were described elsewhere^31^. β-actin (Proteintech, 60008-1-lg) was used as a loading control. According to the size of target proteins, blots were cut for different antibody incubation. The original blots are presented in Supplementary Figures S2–S5.

### RNA isolation and reverse-transcription

Cells were harvested for RNA isolation by using TRI Reagent solution (ThermoFisher) followed with DNase I treatment. cDNA was synthesized from 0.5 μg total RNA with the aid of the Rever Tra Ace qPCR RT Kit (TOYOBO) under instructions provided by the manufacturer.

### Quantitative PCR analysis

mRNA accumulation was analyzed by quantitative PCR by using SYBR Green Realtime PCR master mix (TOYOBO) in Step on plus Real-time PCR system (Applied Biosystems) with indicated primers (as shown in Table 3). GAPDH was used as the normalization control. Relative quantity (RQ) of gene expression was determined with the 2^−ΔΔCt^ method.

**Table 3:** Primers used in this study.

Gene	Accession no	Forward primer (5′–3′)	Reverse primer (5′–3′)
*Mx2*	NM_002463	CAGAGGCAGCGGAATCGTAA	TGAAGCTCTAGCTCGGTGTTC
*TLR2*	NM_003264	ATCCTCCAATCAGGCTTCTCT	GGACAGGTCAAGGCTTTTTACA
*TLR3*	NM_003265	TTGCCTTGTATCTACTTTTGGGG	TCAACACTGTTATGTTTGTGGGT
*TLR8*	NM_138636	ATGTTCCTTCAGTCGTCAATGC	TTGCTGCACTCTGCAATAACT
*TLR9*	NM_017442	CTGCCTTCCTACCCTGTGAG	GGATGCGGTTGGAGGACAA
*RIG I*	NM_014314	AGAAGAGTACCACTTAAACCCAG	TTGCCACGTCCAGTCAATATG
*cGAS*	NM_138441	ACCCAGAACCCTCAAGAC	GAGGCACTGAAGAAAGTATGTC
*MDA5*	NM_022168	AGGAGTCAAAGCCCACCATC	GTGACGAGACCATAACGGATAAC
*DHX9*	NM_001357	CGAACCATCTCAGCGACAAAA	TGAGGTCCATGCTTATTTGCTC
*DHX36*	NM_001114397	GGGTCATGGAGGTAACCGAG	CTCTCCGCTTCCTTGTTCTTC
*IFI16*	NM_005531	AAAGTTCCGAGGTGATGC	TGACAGTGCTGCTTGTGG
*JAK1*	NM_002227	TTGAAAGACAAGACGCTGAT	GATGGCTCGGAAGAAAGG
*JAK2*	NM_004972	CCAGATGGAAACTGTTCGCT	GAGGTTGGTACATCAGAAACAC
*TYK2*	NM_003331	GAGATGCAAGCCTGATGCTAT	GGTTCCCGAGGATTCATGCC
*IFNAR1*	NM_000629	AACAGGAGCGATGAGTCTGTC	TGCGAAATGGTGTAAATGAGTCA
*IFNAR2*	NM_207585	TCATGGTGTATATCAGCCTCGT	AGTTGGTACAATGGAGTGGTTTT
*STAT1*	NM_007315	CAGCTTGACTCAAAATTCCTGGA	TGAAGATTACGCTTGCTTTTCCT
*STAT2*	NM_198332	CCAGCTTTACTCGCACAGC	AGCCTTGGAATCATCACTCCC
*STAT3*	NM_139276	CAGCAGCTTGACACACGGTA	AAACACCAAAGTGGCATGTGA
*IRF9*	NM_006084	GCCCTACAAGGTGTATCAGTTG	TGCTGTCGCTTTGATGGTACT
*MyD88*	NM_001172569	TACAAGGCAATGAAGAAAG	CAAGGCGAGTCCAGAA
*TRIF*	NM_182919	GCCAGCAACTTGGAAATCAGC	GGGGTCGTCACAGAGCTTG
*MAVS*	NM_001206491	CAGGCCGAGCCTATCATCTG	GGGCTTTGAGCTAGTTGGCA
*STING*	NM_198282	TCAGCATTACAACAACCTGCTAC	TTATCCAGGAAGCGAATGTTG
*TBK1*	NM_013254	TGGGTGGAATGAATCATCTACGA	GCTGCACCAAAATCTGTGAGT
*IRF3*	NM_001197128	GCCGAGGCCACTGGTGCATAT	TGGGTCGTGAGGGTCCTTGCT
*IRF7*	NM_004029	GCTGGACGTGACCATCATGTA	GGGCCGTATAGGAACGTGC
*NF-κB*	NM_003998	TAAAGCCCCCAATGCATCCAAC	CCAAATCCTTCCCAGACTCCAC
*IFNα*	NM_000594	AGAGTCACCCATCTCAGCAAG	CACCAGGACCATCAGTAAAGC
*IFNβ*	NM_002176	TTGTGCTTCTCCACTACAGC	CTGTAAGTCTGTTAATGAAG
*IRF1*	NM_002198	ATGCCCATCACTCGGATGC	CCCTGCTTTGTATCGGCCTG
*CH25H*	NM_003956	ATCACCACATACGTGGGCTTT	GTCAGGGTGGATCTTGTAGCG
*TRIM21*	NM_003141	TCAGCAGCACGCTTGACAAT	GGCCACACTCGATGCTCAC
*PKR*	NM_001135652	GCCGCTAAACTTGCATATCTTCA	TCACACGTAGTAGCAAAAGAACC
*IFIT1*	NM_001548	AGAAGCAGGCAATCACAGAAAA	CTGAAACCGACCATAGTGGAAAT
*RSAD2*	NM_080657	TTGGACATTCTCGCTATCTCCT	AGTGCTTTGATCTGTTCCGTC
*BST2*	NM_004335	CACACTGTGATGGCCCTAATG	GTCCGCGATTCTCACGCTT
*GAPDH*	NM_001256799	ATCTTCCAGGAGCGAGATCCCTC	TGAGTCCTTCCACGATACCAAAG
*ICP8*	GU734771.1	GCCTGAAACACACGGTCGTT	ATGGTCGTGTTGGGGTTGAG
*tk*	GU734771.1	AAGGTCGGCGGGATGAG	CGGCCGCGCGATAC

### RNA interference

Gene knockdown was achieved by using siRNA targeting human Mx2, MyD88, STING, DHX9, DHX36, or TLR2. All siRNAs used in this study were purchased from GenePharma and listed with sequences in Table 4. All siRNAs transfections were performed with Lipofectamine RNAiMAX from ThermoFisher according to the manufacturer’s protocol.

**Table 4:** siRNAs used in this study.

Targets	Gene ID	Sequence (5′**–**3′)
*Mx2*	4600	GCAAGGAGCUUCUGGGAUUT
*MyD88*	4615	CCGGCAACUGGAGACACAATT
*STING*	340061	GCCCUUCACUUGGAUGCUUT
*DHX9*	1660	CAGAAGAAGUGGAUUUAAATT
*DHX36*	170506	GAUGCUGCAUAACAUGAAATT
*TLR2*	7097	GCAAGUAUGAACUGGACUUTT
Nontarget (NC)	–	UUCUCCGAACGUGUCACGUTT

### HSV-1 labeled with ethynyl-modified nucleotides (EdU, 5-ethynyl-2′-deoxyuridine) for analysis of HSV-1 genome localization by immunofluorescence assay

Mx2-depleted or mock-depleted A172 cells were inoculated with high MOI EdU genome-labeled T1012G in RPMI 1640 medium containing 0.2% (w/v) BSA and 20 mM HEPES at 4 °C for 1 h, keep constantly for sufficient virus binding. After this period, cells were washed twice with fresh medium and subsequently incubated in fresh medium with 100 μg/ml cycloheximide (Sigma-Aldrich) and 5 μg/ml actinomycin D (Sigma-Aldrich) at 37 °C and 5% CO_2_ for 2 h to allow the viral infection to proceed. At 2 h post-infection, cells were progressed for immunofluorescence assay as follows. Cells were washed twice with PBS, fixed and permeabilized PBS containing 4% (w/v) paraformaldehyde and 0.5% Triton X-100 for 5–10 min at room temperature, and then washed three times with PBS followed by blocking with 3% BSA in PBS for 30 min. Viral genomes were visualized using the Click-iT Plus EdU imaging kit, Alexa Fluor 647 (Invitrogen) according to the manufacturer’s instructions. Nuclear staining was performed with 4′,6-diamidino-2-phenylindole (DAPI) (Invitrogen). Images were captured with a Leica DMi8 microscope system and analyzed with the Image J software.

### Analysis of secreted IFNs via ELISA

The secretion of IFNα, IFNβ and IFNγ in A172 cells with indicated gene knockdown were determined by using Human IFNα (Beyotime), IFNβ (Solarbio) and IFNγ (Beyotime) ELISA kit. Cell supernatant was harvested from treated A172 cells at 24 h post-transfection, subsequently diluted by adding sample solution buffer and performed the assay according to the manufacturer’s instructions. Values were read at 450 nm. The relative quantity of secreted IFNs was shown with OD450 nM. Samples were determined in triplicate.

### FACS analysis

Cells were harvested, washed in cold PBS, and subsequently fixed in cold 70% ethanol for 2 h at 4 °C. After pelleting down, cells were re-suspended in propidium iodide (PI) staining solution from Cell Cycle and Apoptosis Analysis Kit (Beyotime) according to manufacturer’s instructions, followed by dark-incubation at 37 °C for 30 min. Finally, samples were acquired by using a flow cytometer (BD FACS caliber). The data were analyzed with FlowJo 10 software.

### Cell viability analysis

Mx2-depleted and mock-depleted A172 and WIDR cells were infected with oHSV-1 T1012G at an MOI of 0.01, 0.1 MOI and cell viability were analyzed every 24 h over a time course of 72 h by CCK-8 Assays Kit (Cat.C0042, Beyotime) according to manufacturer’s instructions, and the OD450 was detected using the BioTek Epoch.

### Statistical analysis

Graph Pad Prism 8.0 was applied for data analysis and plotting. The data were presented as mean ± SD, representative of three independent experiments performed in triplicate. ns, not significant; *p < 0.05; **p < 0.01; ***p < 0.001.

## Supplementary Information

Supplementary Information.
