# Supplementary material for: High accumulation of Mx2 renders limited multiplication of oncolytic herpes simplex virus-1 in human tumor cells
Source: Sci Rep. 2021 Oct 27;11:21227. doi: 10.1038/s41598-021-00691-y (PMC8551283; doi:10.1038/s41598-021-00691-y)

Figure S1

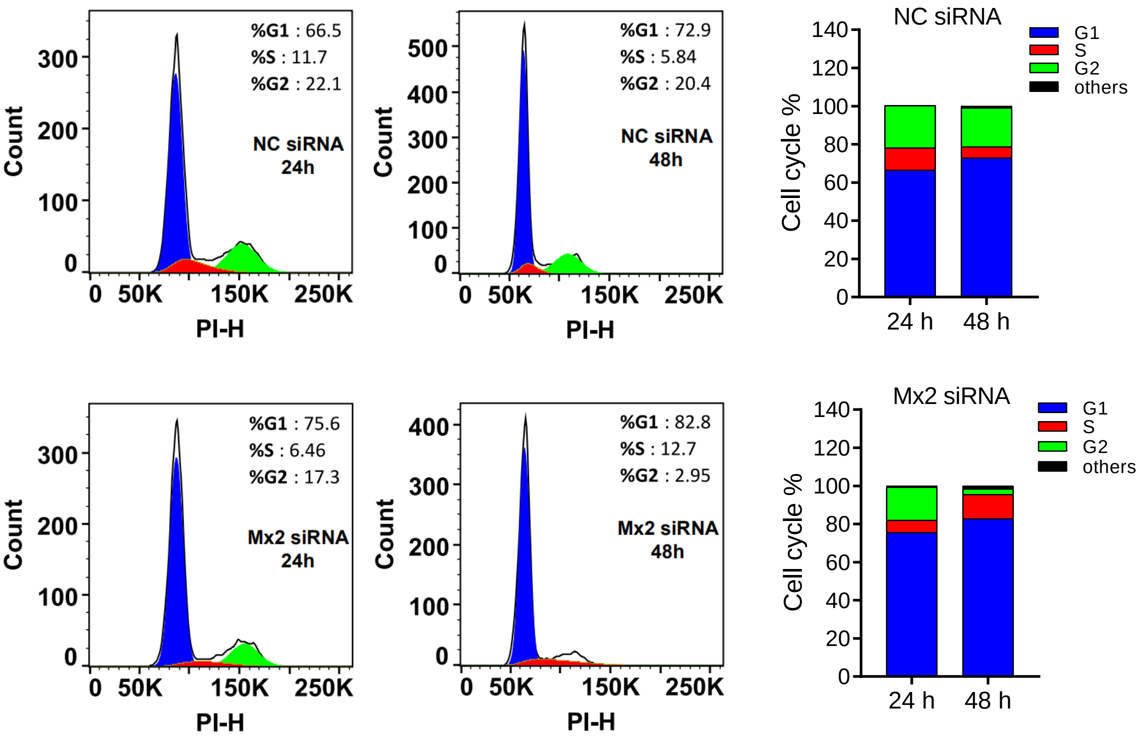

Figure S1. Mx2 depletion perturbs the cell cycle distribution of A172 cells which may facilitate oHSV-1 T1012G lytic replication. Mx2-depleted or mock-depleted A172 cells were harvested at 24 h and 48 h post-transfection, labelled with PI and determined by the FACS analysis as described in Material and Methods. The phases of cell cycle were calculated. Ratios of different phases of cell cycle are shown in the histogram.

Figure S2 : Figure 2A original images

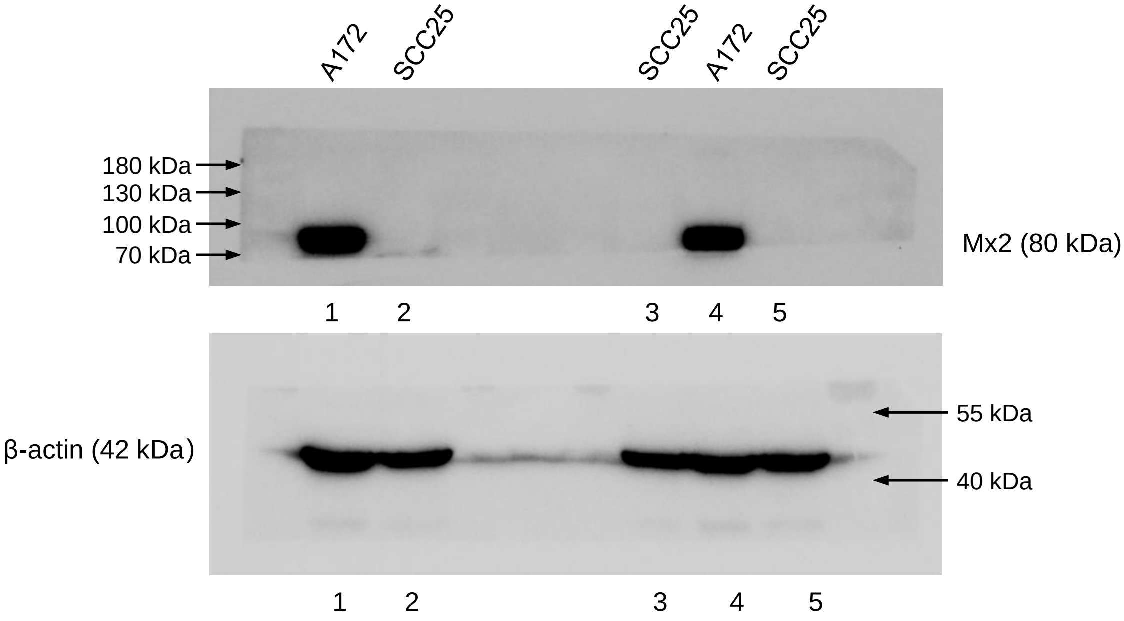

Figure S3： Figure 2D original images

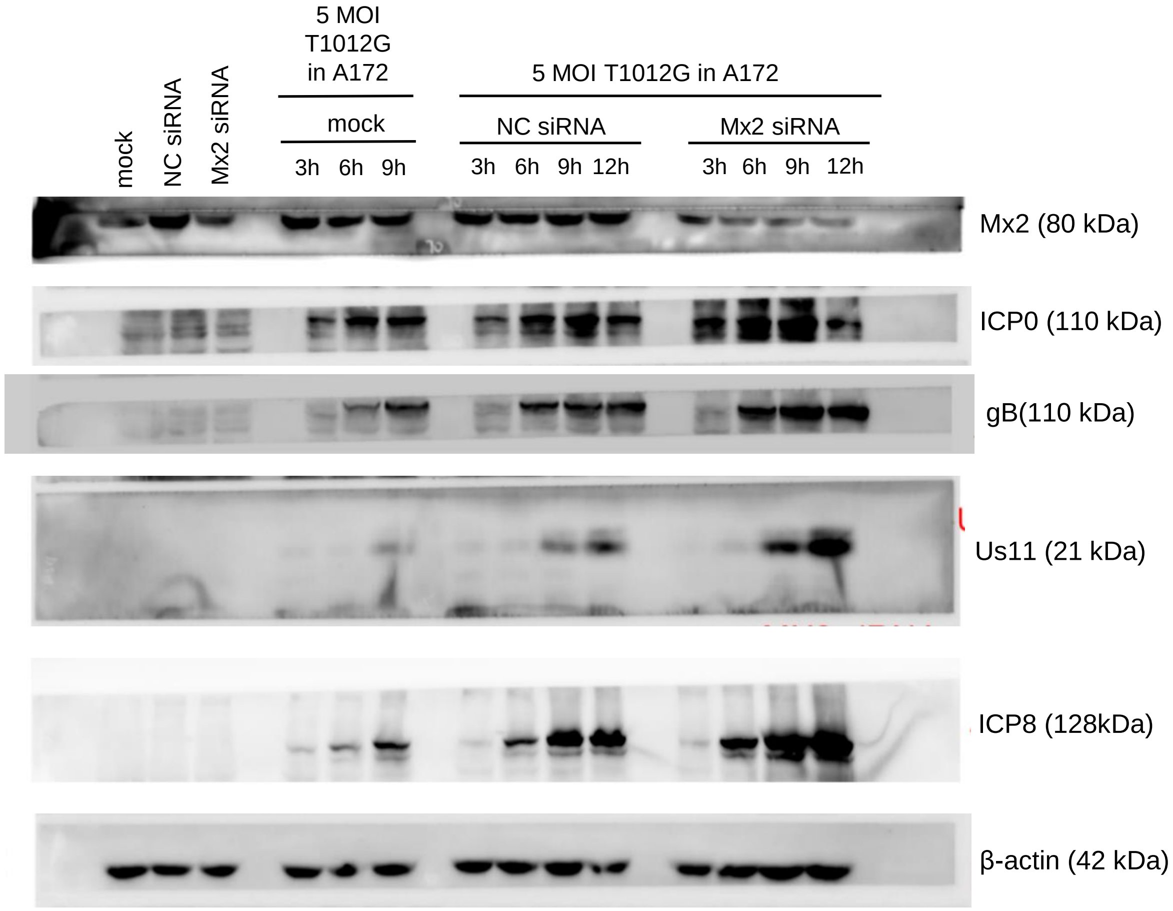

Figure S4 : Figure 4D original images

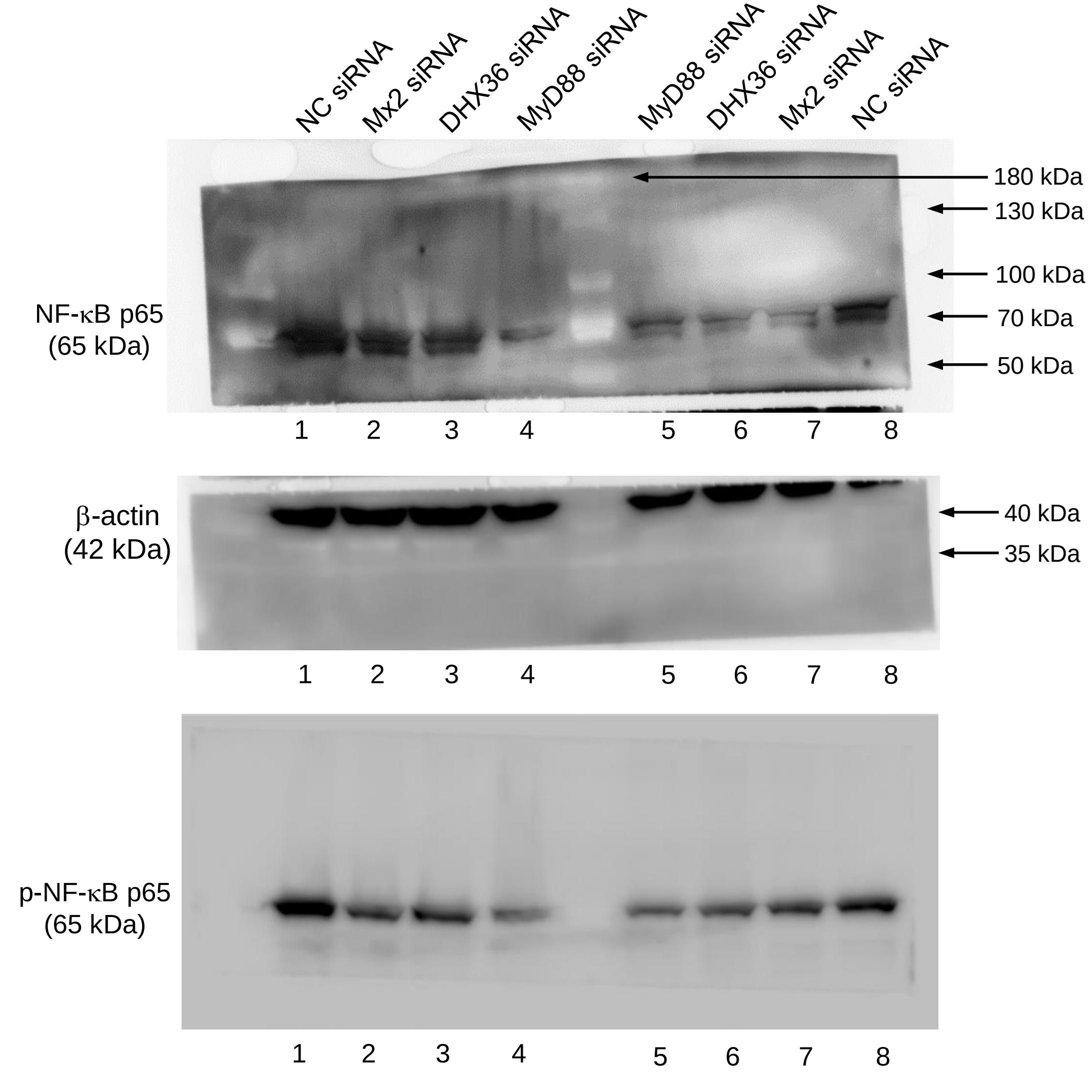

Figure S5： Figure 5I original images

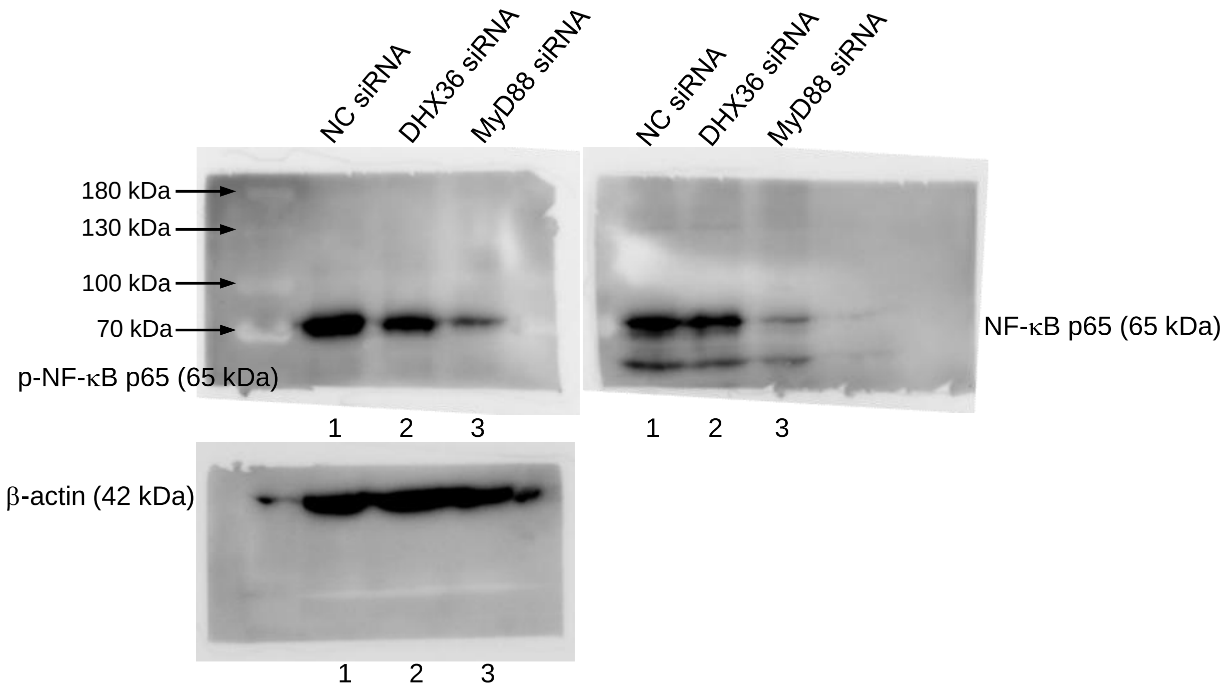

Supplement: Supplementary file 1 — Supplementary Information. [file 41598_2021_691_MOESM1_ESM.pdf]
